# Taxonomy of *Platypterygius campylodon* and the diversity of the last ichthyosaurs

**DOI:** 10.7717/peerj.2604

**Published:** 2016-10-20

**Authors:** Valentin Fischer

**Affiliations:** UR Geology, Université de Liège, Liège, Belgium

**Keywords:** Ichthyosauria, Cretaceous, Extinction, Ophthalmosauridae, Platypterygiinae, Biodiversity, Marine reptiles, Feeding ecology

## Abstract

A complex and confusing taxonomy has concealed the diversity dynamics of Cretaceous ichthyosaurs (Reptilia) for decades. The near totality of Albian-Cenomanian remains from Eurasia has been assigned, by default, to the loosely defined entity *Platypterygius campylodon*, whose holotype was supposed to be lost. By thoroughly examining the Cenomanian ichthyosaur collections from the UK, I redescribe the syntypic series of *Platypterygius campylodon*. This material, along with a handful of other coeval remains, is diagnostic and seemingly differs from the vast majority of Cretaceous remains previously assigned to this taxon. A lectotype for *Platypterygius campylodon* is designated and I reassign this species to *Pervushovisaurus campylodon* nov. comb. The feeding ecology of this species is assessed and conforms to the scenario of an early Cenomanian diversity drop prior to the latest Cenomanian final extinction.

## Introduction

Ichthyosaurs are iconic reptiles of the Mesozoic marine ecosystems that disappeared quite abruptly at the beginning of the Late Cretaceous (Bardet, 1992; Fischer et al., 2016). Understanding of the final chapter of their extensive evolutionary history (Olenekian-Cenomanian, about 157 million years (Bardet, 1992; Motani et al., 2015)) has been impaired by a complex and confusing taxonomy, especially at the supra-specific level. The genus *Platypterygius* is among the most problematic, with no robust phylogenetic definition, no diagnostic features and a biozone spanning the Barremian (‘*Platypterygius*’ *sachicarum*) to the Late Cenomanian (‘*Platypterygius*’ *campylodon*, ‘*Platypterygius kiprijanoffi*’), i.e., 35 million years (Fischer, 2012; Fischer et al., 2014a). Many recent phylogenetic analyses have found the species currently referred to *Platypterygius* to be widely scattered, sometimes within a particular ophthalmosaurid subfamily, Platypterygiinae (Druckenmiller & Maxwell, 2010; Fischer et al., 2012; Fischer et al., 2016; Zverkov et al., 2015). The type species of the genus, *Platypterygius platydactylus*, is phylogenetically isolated from other species currently referred to as *Platypterygius* (Fischer et al., 2016). Moreover, the taxonomy of *Platypterygius* might be biased by ecological convergence (Fischer et al., 2016). Thus, the diversity dynamics of Cretaceous ichthyosaurs cannot be approximated using currently valid genera; the taxonomy of each species needs to critically assessed in isolation and the use of the genus *Platypterygius* should be motivated with respect to the morphology of the type species. Other genus-group names have been used for mid Cretaceous ichthyosaurs in the past but have since been discarded, notably *Myopterygius* Huene, 1922, *Tenuirostria* (Arkhangelsky, 1998) and *Longirostria* (Arkhangelsky, 1998) (Huene, 1922; Arkhangelsky, 1998), adding to the confusion.

A persisting issue in quantifying the diversity and extinction tempo of the last ichthyosaurs is *Ichthyosaurus campylodon* Carter, 1846a, which has been used since its creation (Carter, 1846a; Carter, 1846b) as a bin for nearly all Cretaceous ichthyosaur remains from Eurasia, regardless of their morphology or stratigraphic position. In this brief contribution, I: (i) review the status and morphology of the syntypic material of *Ichthyosaurus campylodon* and other remains from the Cenomanian deposits of the United Kingdom; (ii) rediagnose and designate a lectotype for *I. campylodon*, transfer it to *Pervushovisaurus campylodon* nov. comb and discuss the status and availability of the genus-group taxon *Myopterygius* Huene, 1922; and (iii) assess the ecological diversity of the last ichthyosaurs by the means of a cluster dendrogram analysis of ecomorphological data.

## Material and Methods

**Specimen list**–I surveyed the entire Cenomanian collections of the CAMSM, the RBINS and the NHMUK, but only important specimens are listed here (Table 1). Unlisted remains include centra, undeterminable skeletal fragments and poorly preserved isolated teeth. Specimens from Cambridge Greensand Member (i.e., the base of the West Melbury Marly Chalk Formation, Grey Chalk Subgroup (Hopson, 2005)) have been published elsewhere (Fischer et al., 2012; Fischer et al., 2014b) and are not listed here. I also briefly re-assess the morphology of the specimen used by Broili (1908) to erect the species *Ichthyosaurus kokeni* from the Hauterivian of Germany. This species has been regarded by Huene (1922) as belonging to *Myopterygius*, so it is relevant to discuss its status and morphology here.

**Table 1:** Important West Melbury Marly Chalk Formation specimens studied here.

Specimen	Material	Assignation	Locality
CAMSM B20643	Tooth	Platypterygiinae indet. (holotype of *I. angustidens*= nomina nuda Fischer et al. (2014b))	Hunstanton
CAMSM B20644	Tooth	*Pervushovisaurus campylodon* (syntype, Carter’s series)	Cambridge area
CAMSM B20645	Tooth	Platypterygiinae indet. (syntype, Carter’s series)	Cambridge area
CAMSM B20646	Tooth	*Pervushovisaurus campylodon* (syntype, Carter’s series)	Cambridge area
CAMSM B20647	Tooth	*Pervushovisaurus campylodon* (syntype, Carter’s series)	Cambridge area
CAMSM B20648	Tooth	*Pervushovisaurus campylodon* (syntype, Carter’s series)	Cambridge area
CAMSM B20649	Tooth	*Pervushovisaurus campylodon* (syntype, Carter’s series)	Cambridge area
CAMSM B20650	Tooth	*Pervushovisaurus campylodon* (syntype, Carter’s series)	Cambridge area
CAMSM B20651	Tooth	*Pervushovisaurus campylodon* (syntype, Carter’s series)	Cambridge area
CAMSM B20652	Tooth	*Pervushovisaurus campylodon* (syntype, Carter’s series)	Cambridge area
CAMSM B20653	Tooth	*Pervushovisaurus campylodon* (syntype, Carter’s series)	Cambridge area
CAMSM B20654	Tooth	*Pervushovisaurus campylodon* (syntype, Carter’s series)	Cambridge area
CAMSM B20655	Tooth	*Pervushovisaurus campylodon* (syntype, Carter’s series)	Cambridge area
CAMSM B20656	Tooth	*Pervushovisaurus campylodon* (syntype, Carter’s series)	Cambridge area
CAMSM B20657	Tooth	*Pervushovisaurus campylodon* (syntype, Carter’s series)	Cambridge area
CAMSM B20658	Tooth	*Pervushovisaurus campylodon* (syntype, Carter’s series)	Cambridge area
CAMSM B20659	Partial rostrum	*Pervushovisaurus campylodon* (syntype, Carter’s series)	Cambridge area
CAMSM B20671a	Rostrum	*Pervushovisaurus campylodon*	Barrington
CAMSM B75736	Atlas-axis	Ichthyosauria indet.	Cambridge area
CAMSM B42257	Centrum	Ichthyosauria indet.	Hunstanton
CAMSM TN282	Rostrum	*Pervushovisaurus campylodon*	(chalky part of the Cambridge Greensand Member, Cambridge area)
CAMSM TN283	Rostrum	Platypterygiinae indet.	(chalky part of the Cambridge Greensand Member, Cambridge area)
CAMSM unnumbered	Humerus (HM1 morphotype of Fischer et al. (2014b))	Platypterygiinae indet.	Cambridge area
NHMUK 5648	Teeth	Platypterygiinae indet.	?
NHMUK 33294 partim	Teeth	Platypterygiinae indet.	Isleham, Cambridgeshire
NHMUK 41367	Anterior tip of rostrum	Platypterygiinae indet.	?
NHMUK 41895	Anterior tip of rostrum	Platypterygiinae indet.	?
NHMUK R13	Teeth	Platypterygiinae indet.	?
NHMUK R49	Teeth	Platypterygiinae indet.	Lyden Spout, Folkestone
NHMUK R2335	Rostrum	Platypterygiinae indet.	?
NHMUK R2385	Fragmentary rostrum	Platypterygiinae indet.	?

**Late Cretaceous ichthyosaur feeding guilds–**I updated the ecological dataset of Fischer et al. (2016) to assess the feeding guilds and ecological diversity of the last ichthyosaurs. This dataset is composed of a series of ecologically-relevant measurements and ratios: absolute tooth size, crown shape (height/basal diameter), crown height relative to basioccipital diameter, relative symphysis length, relative snout depth, absolute aperture of the sclerotic ring and quantification of tooth wear. I have added novel data on the symphysis of ‘*Platypterygius*’ *sachicarum* (E Maxwell pers. comm., 2016) corrected an erroneous value on the symphysis of the ‘*Platypterygius*’ *hercynicus* and added *Pervushovisaurus campylodon* to the dataset. The details of the specimens used and explanation for each character is given in Supplemental Information 1; the dataset itself is available as Data S1. As in the original publication, I submitted this data set to a cluster dendrogram analysis in R using the Ward method. Data were scaled to have equal variances and transformed to a Euclidean distance matrix before clustering. Because the data is restricted to ecologically relevant measurements and with a strong emphasis on Cretaceous forms, the resulting dataset is small and contain a non-negligible proportion of missing values (39%), which renders usual bootstrapping methods inadequate. To cope with this issue, I assessed the statistical support of our cluster using the “Approximately Unbiased *P*-value” method of the pvclust v2.0–0 package (Suzuki & Shimodaira, 2015) in R. This method employs multiscaled bootstrapping: instead of simply bootstrapping the dataset, it creates multiple datasets that are smaller, equal and larger than the original dataset. I ran it from 0.5 times to 5 times the size of the original dataset, with 0.1 increments and 10,000 bootstrap per increment.

**Nomenclatural acts–**The electronic version of this article in Portable Document Format (PDF) will represent a published work according to the International Commission on Zoological Nomenclature (ICZN), and hence the new names contained in the electronic version are effectively published under that Code from the electronic edition alone. This published work has been registered in ZooBank. Zoobank does not currently support nomenclatural acts that do not establish new taxa (e.g., nov. comb.), so the specific nomenclatural acts of this paper cannot be entered in Zoobank for the time being. The ZooBank LSIDs (Life Science Identifiers) can be resolved and the associated information viewed through any standard web browser by appending the LSID to the prefix “http://zoobank.org/”. The LSID for this publication is: urn:lsid:zoobank.org:pub:019DACEA-EBBE-4FAE-B885-3A9E5B1E1315. The online version of this work is archived and available from the following digital repositories: PeerJ, PubMed Central and CLOCKSS.

## History and Status of *Platypterygius Campylodon*

Carter (1846a) established the name *Ichthyosaurus campylodon* in a conference abstract. His initial description is based on an articulated rostrum with numerous teeth that he described in a paper the same year (Carter, 1846b). In that paper, he figured two teeth and made clear that his collection contained several specimens, coming from both the base (Cambridge Greensand Member) and the rest of the West Melbury Marly Chalk Formation. The Cambridge Greensand member mixes earliest Cenomanian specimens with reworked fossils from the Late Albian of the underlying Gault Formation (Hopson, 2005; Fischer et al., 2014b). It is therefore difficult to know which particular specimen was used to establish the species in Carter’s conference abstract, but relevant information can be extracted from the specimens from his collection, which are now housed in the Sedgwick Museum of the University of Cambridge, UK (CAMSM).

Huene (1922) assigned *Ichthyosaurus campylodon* to the genus *Myopterygius* Huene, 1922 and created another genus, *Platypterygius* Huene, 1922 for reception of a single species from the Lower Aptian of Germany, *Platypterygius platydactylus* (Broili, 1907). McGowan (1972) then transferred all species belonging to *Myopterygius* to *Platypterygius*. He choose *Platypterygius* over *Myopterygius* as the single valid Cretaceous ichthyosaur genus “*Because* platydactylus *is the best known species, the genus* Platypterygius *is the most appropriate*” (McGowan, 1972: 18). Since Carter’s and McGowan’s publications, an overwhelming amount of Cretaceous ichthyosaur remain from Eurasia has been referred to *Platypterygius campylodon*, mostly by default (e.g., Kiprijanoff, 1881; Kiprijanoff, 1883; Sauvage, 1882; Delair, 1960; Buffetaut, 1977; Buffetaut et al., 1981; Buffetaut, Tomasson & Tong, 2003). Some remains were referred to the species *Platypterygius kiprijanoffi* (Romer, 1968; Bardet, 1989), but these were subsequently assigned to as *Platypterygius campylodon* by McGowan & Motani (2003).

Currently, *Platypterygius campylodon* is a vague entity with no clear-cut morphology nor any valid diagnostic feature, itself included in a poorly defined genus. As a matter of fact, the only diagnostic feature proposed by McGowan & Motani (2003) for *Platypterygius campylodon* is the probable presence of an “*External longitudinal groove […] along the length of the rostrum and mandible*” (=fossa praemaxillaris/dentalis); such sulcus is actually present in all neoichthyosaurians I have examined so far. With no holotypic or syntypic material clearly identified as such and no diagnostic feature, this species had to be considered as a nomen dubium.

**Figure 1:**
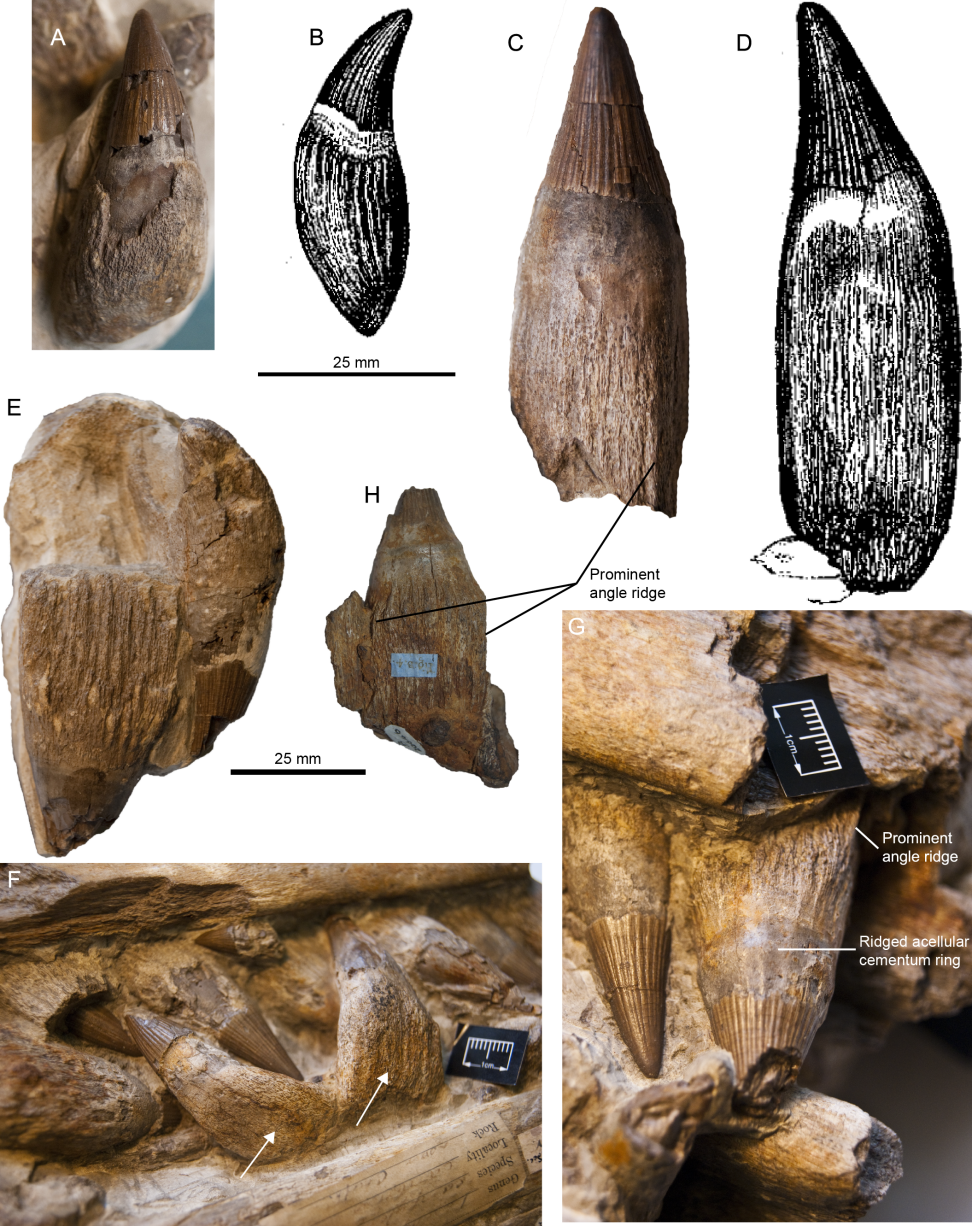
Syntypic material of *Pervushovisaurus campylodon* (Carter, 1846a). (A) CAMSM B20645 a posterior tooth likely to be the one figured by Carter (1846b), which is reproduced in (B). This tooth cannot be unambiguously referred to *I. campylodon* and is regarded as Platypterygiinae indet. (C) CAMSM B20644, a large mid-snout tooth, likely to be the one figured by Carter (1846b), which is reproduced in (D). (E–G) Teeth from CAMSM B20659, a partial rostrum; this specimen was figured by Owen (1851) (Pl. XXV) and is here selected as the lectotype. (E) Small posterior tooth from CAMSM B20659. (F) Mid-snout dentary teeth from CAMSM B20659. White arrows indicate curved tooth roots in the lower jaw, considered by Carter (1846b) (and subsequent authors) as a diagnostic feature. This feature is here regarded as doubtful and appears to be diagenetic. (G) Mid-snout premaxillary teeth from CAMSM B20659. (H) Small mid-snout tooth (CAMSM B20646) illustrating the sharp angle ridges on the root.

McGowan & Motani (2003) attempted to solve this issue. They regarded the specimen SMC B20644 (=CAMSM B20644), “a 60-cm rostral fragment”, as the presumed holotype for *Ichthyosaurus campylodon*, mainly because its size matched the length given by Carter (“*more than 2 feet*”, p7 in Carter, 1846b). But there are several issues with that decision. Firstly, CAMSM B20644 is not a 2 feet-long rostrum but an isolated tooth from Carter’s *Ichthyosaurus campylodon* collection; this tooth actually seems to be the tooth figured by Carter (Figure a in Carter (1846b: 6); see Fig. 1), as already suggested by Delair (1960). McGowan & Motani (2003: 120) actually figured a portion of another specimen, CAMSM B20671. CAMSM B20671 is actually more complete than figured in McGowan & Motani (2003) and has diagnostic features (see below), but that specimen is 790 mm, i.e., 2.59 feet long. CAMSM B20671 preserves the tip of both the rostrum and the mandible, whereas Carter clearly stated that the specimen he described lacked these parts (Carter, 1846b: 7). Moreover, CAMSM B20671 is from Barrington quarry and the date written on the specimen is 1881, 35 years after Carter’s original descriptions. While this date may be the acquisition date by the museum, all specimens from Carter’s collection have a green label glued on them containing “*Presented by J. Carter Fsq.ES.G*” and CAMSM B20671 lacks such a label. Actually, there is not a single 2 feet long rostrum in the CAMSM that bears such label. Delair (1960) listed B.20644-59 C.U.M. B.58379-82 C.U.M (CAMSM B20644_59 and CAMSM B58379_82) as “types” of *Ichthyosaurus campylodon*, but without any justification or argument for this a posteriori designation. CAMSM B58379_82 are phosphatized teeth from the Cambridge Greensand Member and are therefore reworked from the upper part of the Gault formation (Upper Albian, see Fischer et al. (2014b) for a treatment of these remains), while CAMSM B20644_59 are teeth from the unreworked part of the West Melbury Marly Chalk Formation and are Early Cenomanian in age (Hopson, 2005). This, again, contradicts Carter’s account (1846a), which specifically discussed the morphology of a partial rostrum with associated teeth.

**Figure 2:**
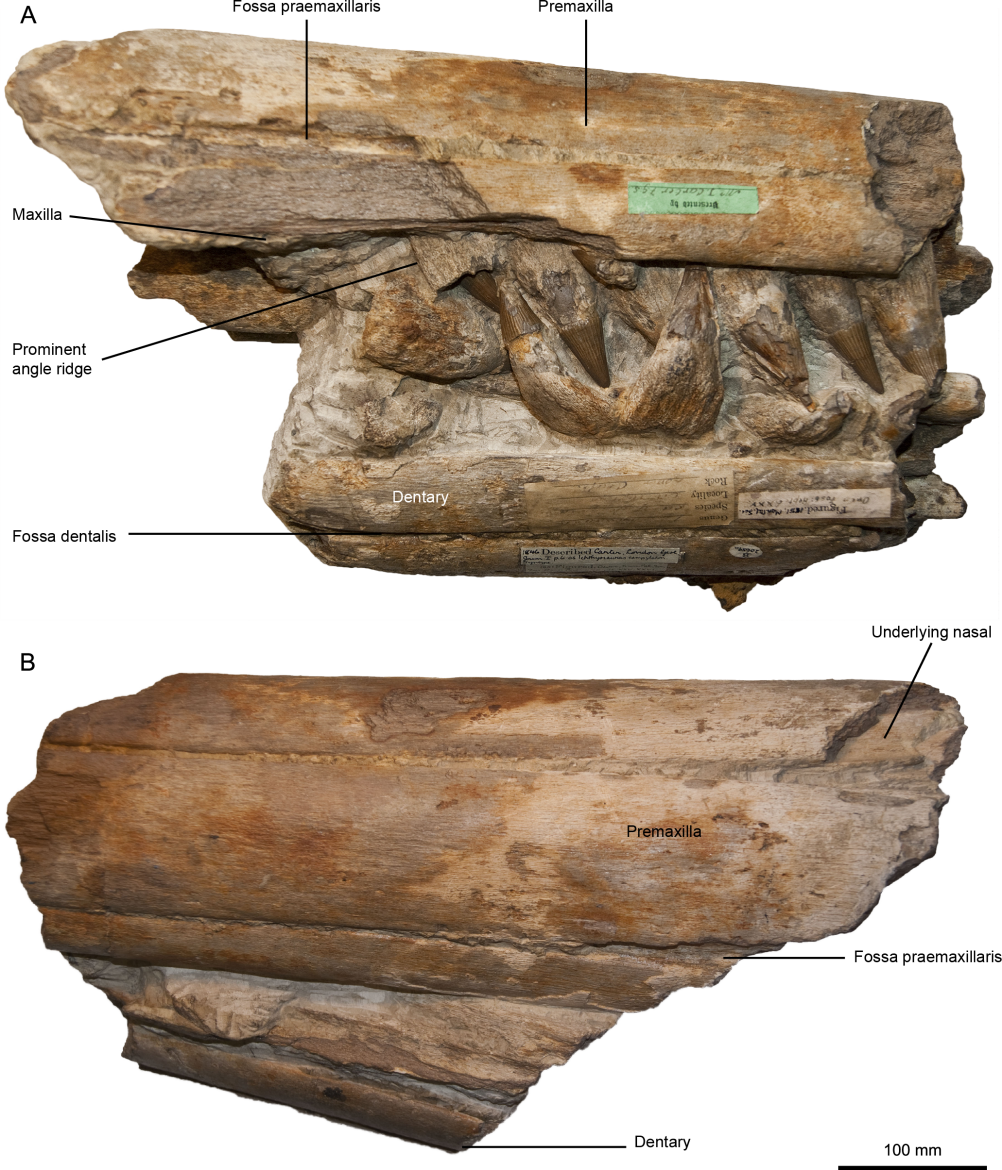
Designated lectotype for *Pervushovisaurus campylodon* (Carter, 1846a), CAMSM B20659. (A) Mid-snout fragment in right lateral view, showing the diagenetically deformed dentary teeth. (B) Same fragment in dorsolateral view.

There are other large rostra lacking the anterior tip in the CAMSM, but these lack most of their teeth, so these do not match Carter’s description either. However, a fragmentary rostrum identified as belonging to Carter’s collection (CAMSM B20659) possesses markedly curved teeth (Figs. 1 and 2). This is probably the material used by Carter to define the species (‘campylodon’ meaning ‘bent tooth’), as the mandibular teeth appear markedly recurved compared to the (pre)maxillary teeth, matching Carter’s description. Owen (1851: Pl. XXV) figured this specimen, which seemed to include a much longer portion of the rostrum at that time, thus possibly extending up to two feet. By all means, all articulated rostra discussed by Carter and the teeth he likely figured belong to the unreworked part of the West Melbury Marly Chalk Formation, not from the Late Albian of the Gault Formation. While the presumed holotype of *Ichthyosaurus campylodon* cannot be located with certainty, there is an abundant material from the Grey Chalk Subgroup in Carter’s Collection, and some specimens are clearly identified as being “*syntypes*”: CAMSM B20659 and a series of teeth CAMSM B20644_58, containing the ones likely figured by Carter (1846b) (Figs. 1 and 2, Fig. S1). This material can thus serve as a nucleus to redefine *Ichthyosaurus campylodon,* assess its supraspecific attribution and evaluate the diversity of the last European ichthyosaurs.

## Systematic Palaeontology

**Table:** 

ICHTHYOSAURIA Blainville, 1835
THUNNOSAURIA Motani, 1999
OPHTHALMOSAURIDAE Baur, 1987
PLATYPTERYGIINAE Arkhangelsky, 2001 (sensu Fischer et al., 2012)

***PERVUSHOVISAURUS*Arkhangelsky, 1998**

**Type species**–*Pervushovisaurus bannovkensis* (Arkhangelsky, 1998)

**Additional included species**–*Pervushovisaurus campylodon* (Carter, 1846a; Carter, 1846b) nov. comb.

**Emended diagnosis** (from Fischer et al., 2014a) **—**Platypterygiine ophthalmosaurid characterized by the following autapomorphies (those marked by an asterisk cannot be assessed in the material referred to *Pervushovisaurus campylodon*): presence of foramina along the ventral premaxillary–maxillary suture*; presence of a semi-oval foramen on the lateral surface of the premaxilla, anteroventral to the external naris*; presence of lateral ridges on the maxilla*; presence of wide supranarial ‘wing’ of the nasal (a similar structure, although much smaller, is present in ‘*Platypterygius*’ *australis* and *Acamptonectes densus*)* (see Kear, 2005; Fischer et al., 2012, respectively); robust splenial markedly protruding from the external surface of the mandible; root with quadrangular cross-section, with the cementum forming prominent 90° angles.

*Pervushovisaurus* is also characterized by the following unique combination of features: secondarily closed naris surrounded by foramina* (as in ‘*Platypterygius*’ *sachicarum* and ‘*Platypterygius*’ *australis* (see Paramo, 1997; Kear, 2005, respectively), and in *Simbirskiasaurus birjukovi*, although the ‘anterior’ naris is still present in this taxon (Maisch & Matzke, 2000; Fischer et al., 2014a)); elongated anterior process of the maxilla, reaching anteriorly the level of the nasal (unlike in *Aegirosaurus leptospondylus*, *Sveltonectes insolitus* and *Muiscasaurus catheti*) (Bardet & Fernández, 2000; Fischer et al., 2011a; Maxwell et al., 2015, respectively); rostrum straight (unlike in ‘*Platypterygius*’ *americanus*, ‘*Platypterygius*’ *sachicarum*, ‘*Platypterygius*’ *australis* and possibly *Muiscasaurus catheti*, where it is slightly curved anteroventrally Romer, 1968; Paramo, 1997; Kear, 2005; Maxwell et al., 2015, respectively); straight, non-recurved tooth crowns (unlike in *Sveltonectes insolitus*, *Muiscasaurus catheti)* (Fischer et al., 2011a; Maxwell et al., 2015, respectively).

**Stratigraphic range**–Early-middle Cenomanian, Late Cretaceous.

**Geographic range**–Europe–western Russia.

**Figure 3:**
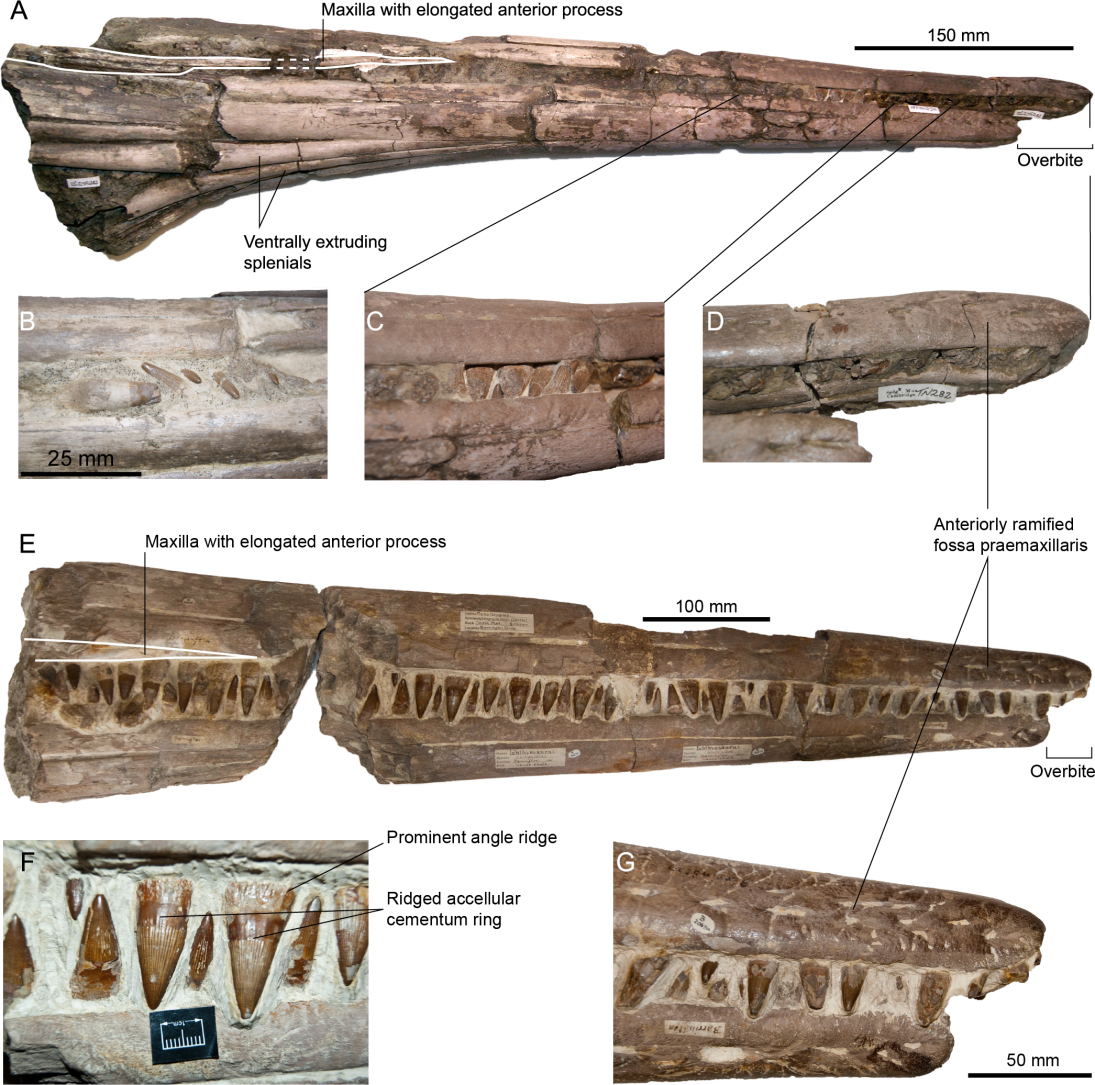
Rostra referred to *Pervushovisaurus campylodon* (Carter, 1846a). (A–D) CAMSM TN282, a partial rostrum possibly from a juvenile specimen. (A) Ventrolateral view. (B–C) Details of the teeth. (D) Detail of the premaxillary overbite. (E–F) CAMSM B20671a, a partial rostrum of a large specimen. (E) Lateral view. (F) detail of the mid-snout teeth. (G) Detail of the premaxillary overbite.

***PERVUSHOVISAURUS CAMPYLODON* (Carter, 1846a) nov. comb. Figs. 1–3**

**Table:** 

1846a *Ichthyosaurus campylodon*—Carter
v 1846b *Ichthyosaurus campylodon*—Carter
v 1851 *Ichthyosaurus campylodon*—Owen
1922 *Myopterygius campylodon*—Huene
v 1960 *Myopterygius campylodon*—Delair
v 1972 *Platypterygius campylodon*—McGowan
v 2003 *Platypterygius campylodon*—McGowan & Motani

**Syntype series and lectotype**–CAMSM B20644, CAMSM B20646 to CAMSM B20658, a series of teeth (including a tooth likely figured in Carter, 1846b); CAMSM B20659, a partial rostrum, all from the West Melbury Marly Chalk Formation (Early Cenomanian), Cambridgeshire, UK. CAMSM B20659 is here formally designated as the lectotype (ICZN Articles 72.1.1, 73 and 74). Other specimens of the series (CAMSM B20644, CAMSM B20646 to CAMSM B20658) are thus designated as paralectotypes.

**Referred specimens**–CAMSM B20671a and CAMSM TN282, two partial rostra from the upper (chalky) part of the Cambridge Greensand Member (earliest Cenomanian), Cambridgeshire, UK (the specific locality of CAMSM B20671a is recorded: Barrington); NHMUK 33294 partim, a nearly complete tooth lacking the apex and the distal part of the root, from the West Melbury Marly Chalk Formation at Isleham, Cambridgeshire, UK; NHMUK R49, a series of articulated teeth from the West Melbury Marly Chalk Formation at Lydden Spout, Folkestone, UK.

**Emended diagnosis**–*Pervushovisaurus campylodon* characterized by the following autapomorphy: slight overbite (3–4 cm). *Pervushovisaurus campylodon* is also characterized by the following unique combination of features: crown with rugose texture (shared with *Aegirosaurus* sp., ‘*Platypterygius*’ *hercynicus* and ‘*Platypterygius*’ sp. specimens from France and UK Fischer et al., 2011b; Fischer et al., 2014b; Fischer, 2012); acellular cementum ring of mid-snout teeth can possess shallow apicobasal ridges and furrows (shared with ‘*Platypterygius*’ *australis*) (Maxwell, Caldwell & Lamoureux, 2011).

**Type horizon and locality–**Lower Cenomanian of the Grey Chalk Subgroup, Upper Cretaceous. Cambridge area, Cambridgeshire, UK.

**Remarks**–The designated lectotype (CAMSM B20659), many teeth from rest of the syntypic series (CAMSM B20644, CAMSM B46_58) and the referred rostra (CAMSM B20671a, CAMSM TN282) each exhibit diagnostic features (Table 2). This material can be combined into a morphologically and spatiotemporally homogenous series that is distinguishable from the other ophthalmosaurid ichthyosaurs for which rostral and dental features have been reported.

**Table 2:** Distribution of the diagnostic features of *Pervushovisaurus campylodon* among the available specimens.

Osteological feature	Carter’s teeth (CAMSM B20644, CAMSM B46_58a)	Syntypic rostrum (CAMSM B20659)	Referred rostra (CAMSM B20671a, CAMSM TN282)
Long maxilla	?	Y	Y
Prominent root angles	Y	Y	Y
Rugose enamel	Y	Y	Y
Ridged acellular cementum ring	Y	Y	Y
Thickened splenial	?	?	Y
Straight rostrum	?	?	Y
Overbite	?	?	Y

CAMSM TN283, a large rostrum also originating from the Grey Chalk Subgroup, Cambridgeshire, closely resembles CAMSM B20671a and CAMSM TN282, but the autapomorphies of *Pervushovisaurus campylodon* cannot be evaluated unambiguously in this specimen; it is thus referred to as Platypterygiinae indet. A series of teeth and tooth bearing elements from the West Melbury Marly Chalk Formation collections of the NHMUK (NHMUK R1916, NHMUK R2335, NHMUK R2339, NHMUK 41895, NHMUK 47233, NHMUK 49911, NHMUK 52819) are, similarly, compatible with *Pervushovisaurus campylodon* in terms of tooth crown shape and size, and maxilla anterior extension but cannot be unambiguously referred to as *Pervushovisaurus campylodon*; these are thus referred to as Platypterygiinae indet. as well.

**PLATYPTERYGIINAE indet**

**Referred specimen** (see Table 1 for details)–CAMSM B20645; CAMSM TN283; CAMSM unnumbered; NHMUK R13; NHMUK R49; NHMUK R1916; NHMUK R2335; NHMUK R2339; NHMUK R2385; . NHMUK 5648; NHMUK 33294 partim; NHMUK 41367; NHMUK 41895, NHMUK 47233, NHMUK 49911, NHMUK 52819.

**Note**—CAMSM B20645 is a tooth is part of the type series of *Ichthyosaurus campylodon*, but lacks the diagnostic features of *Pervushovisaurus* and *Pervushovisaurus campylodon*; it is thus regarded as Platypterygiinae indet.

**Table:** 

**OPHTHALMOSAURINAE** Baur, 1887 sensu Fischer et al. (2012)
**OPHTHALMOSAURINAE indet**.

**Referred specimen**–Unknown specimen number, holotype of *Ichthyosaurus kokeni* (Broili, 1908).

**Stratigraphy**–Grodischter Schichten; middle and upper ‘Neocomian’.

**Location–**Vicinity of Hannover, Germany.

**Synonymy**

1908 *Ichthyosaurus kokeni* Broili: 432

**Discussion—***Ichthyosaurus kokeni* is based on a basioccipital, and a partial humerus, a paddle element and several centra from the “Grodischter” schist, considered as Hauterivian in age by Broili (1908). A peripheral groove surrounds the condyle of the basioccipital, as in ophthalmosaurine ophthalmosaurids (Fischer et al., 2012). The humerus has three facets and the ulnar facet appears deflected posterolaterally with respect to the sagittal plane, further indicating ophthalmosaurine affinities (Fischer et al., 2012). The material lacks autapomorphies or unique combination of features and should therefore be regarded as a nomen dubium, assigned to Ophthalmosaurinae indet.

## Description and Comparison of *Pervushovi- saurus Campylodon*

**Premaxilla (CAMSM B20659; CAMSM B20671a; CAMSM TN282; Figs. 2 and 3)**–The premaxilla is markedly elongated and has a semi-circular cross-section. Fossa praemaxillaris is a deep and continuous sulcus that is segmented anteriorly in a series of aligned foramina. As in *Suevolevithan* (Maisch, 2001) and some other Cretaceous ichthyosaurs (V Fischer, pers. obs. on unpublished material from the Albian of France, 2012), a complex network of the shallow grooves radiates from these foramina and textures the lateral surface of the premaxilla. In the anterior third of the rostrum, the dental groove is slightly constricted between functional teeth, forming subtle pseudo-alveoli. The labial wall of the dental groove then becomes straight and thickens posteriorly. The premaxilla forms a slight overbite (4–5 cm), a unique feature among ophthalmosaurids. This overbite is genuine because premaxillary and dentary teeth are still tightly interlocked in the anterior part of the rostrum in these specimens (CAMSM TN282, CAMSM B20671a).

**Maxilla (CAMSM B20659; CAMSM B20671a; CAMSM TN282; Figs. 2 and 3)**—The anterior process of the maxilla is elongated and its external extent reaches the level of emergence of the nasal, as in many platypterygiines, except *Aegirosaurus, Sveltonectes*, and *Muiscasaurus* (Romer, 1968; Kirton, 1983; Bardet & Fernández, 2000; Sirotti & Papazzoni, 2002; Fischer et al., 2011a; Fischer et al., 2011b; Maxwell et al., 2015) (note that Sirotti & Papazzoni (2002) interpreted the rostrum upside-down).

**Dentary (CAMSM B20659; CAMSM B20671a; CAMSM TN282; Fig. 3)**–The dentary is elongated, semi-circular and slightly deeper than the premaxilla. Fossa dentalis is narrow and ends anteriorly as a series of aligned foramina. Like in the premaxilla, the labial wall of the dental groove is constricted between functional teeth in the anterior third of the dentary. It is straight, unlike in some other platypterygiines (‘*Platypterygius*’ *australis*, ‘*Platypterygius*’ *americanus* and ‘*Platypterygius*’ *sachicarum* (Romer, 1968; Paramo, 1997; Kear, 2005)). The dentary is reduced anteriorly, creating an overbite.

**Splenial (CAMSM B20671a; CAMSM TN282; Fig. 3)**—The symphysis is 535 mm long in CAMSM TN282. The splenials are markedly thickened ventrally near the end of the symphysis, similar to the condition seen in *Pervushovisaurus bannovkensis* and regarded as one of the autapomorphies of this taxon (Fischer et al., 2014a).

**Dentition (CAMSM B20644; CAMSM B20646_58; CAMSM B20659; CAMSM B20671a; CAMSM TN282; Figs. 1–3)**—The crown is conical, robust, and covered by rugose enamel (as in *Aegirosaurus* sp., ‘*Platypterygius*’ * hercynicus* and *Platypterygius* sp. Fischer et al., 2011b; Fischer et al., 2014b; Fischer, 2012, respectively). Smaller specimens like CAMSM TN282 tend to have slenderer teeth. The acellular cementum ring is ridged on large teeth, but only apically, as in ‘*Platypterygius*’ *australis* (Maxwell, Caldwell & Lamoureux, 2011). The root possesses markedly flattened surfaces (mostly anterior and posterior ones); the root cement forms protruding ridges in between these facets, forming prominent and sharp ridges with a 90° angle cross-section, as in *Pervushovisaurus bannovkensis* (see Fischer et al., 2014a). This marks a sharp increase of the ‘diameter’ of the tooth, unlike in ‘*Platypterygius*’ *hercynicus* and many other isolated teeth from the Cambridge Greensand Member (Kuhn, 1946; Fischer et al., 2014b), where the diameter increases gradually. Numerous apicobasal ridges texture the labial and lingual surfaces of the root. Some of the dentary teeth of Carter’s syntype (CAMSM B20659) are markedly bent inwardly, which lead Carter to propose the name “*campylodon*” for reception of this material. However, slightly bent teeth are commonly encountered in many ichthyosaur specimens (Sollas, 1916; McGowan & Motani, 2003). While the dental grooves of the dentary appear indeed slightly oblique with respect to the sagittal plane, the strong bend appears here to result from diagenetic compression. I consider this feature as poorly diagnostic, and only very few isolated teeth exhibit a similar curvature of the root.

## Cluster Dendrogram Results

**Figure 4:**
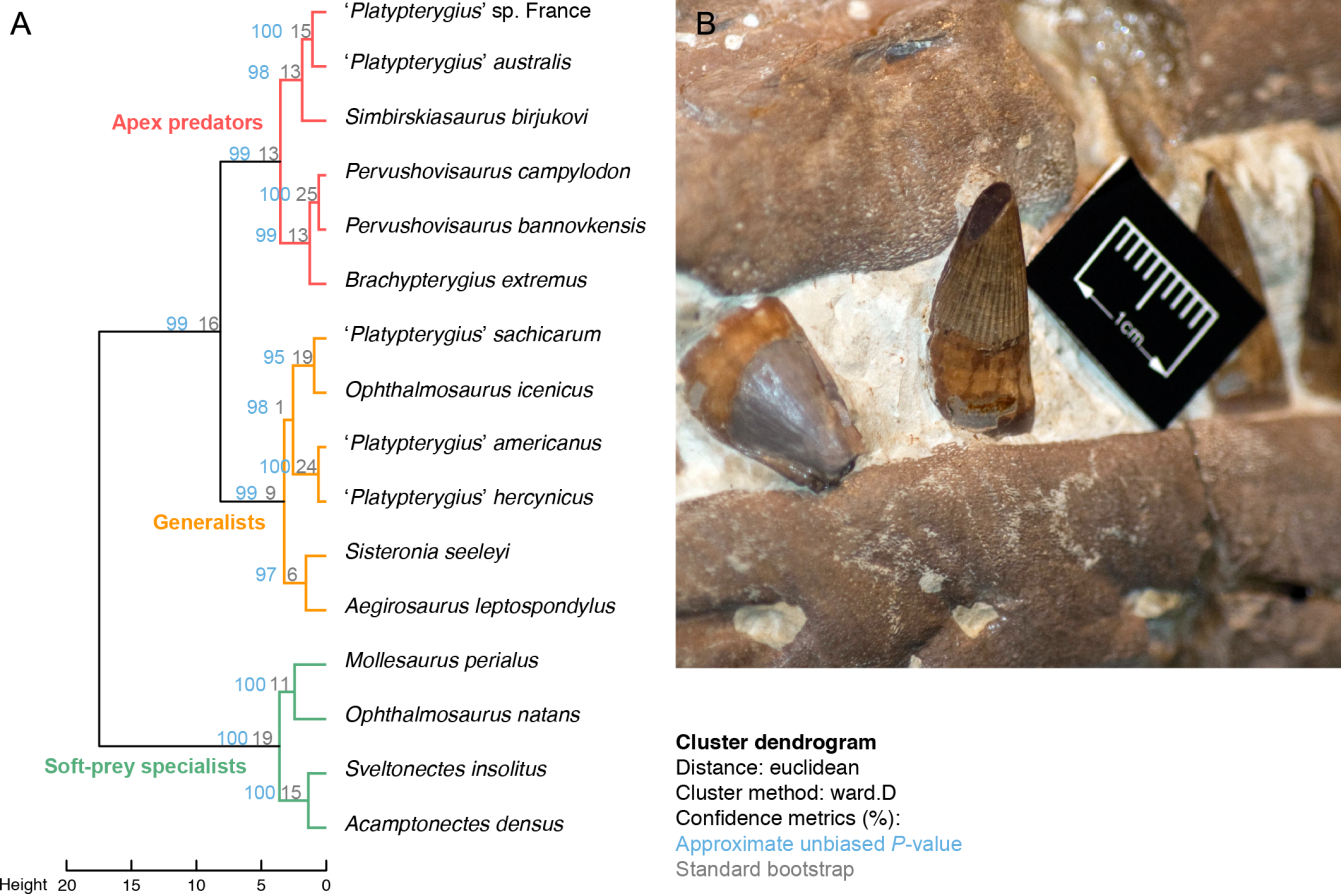
Feeding ecology of the last ichthyosaurs. (A) Cluster dendrogram resulting from the analysis of the ecomorphological dataset and showing separation of three main guilds. (B) Detail of spalled and subsequently polished apex in CAMSM TN283 (Platypterygiinae indet., closely resembling *Pervushovisaurus campylodon*).

The cluster dendrogram analysis resulted in a similar groupings than in Fischer et al. (2016). One exception is the displacement of ‘*Platypterygius*’ *hercynicus* and ‘*Platypterygius*’ *americanus*, two taxa with slightly smaller crowns, to the Generalist guild, from the Apex Predator guild (Fig. 4). These taxa remain clustered with ‘*Platypterygius*’ *australis* and *Brachypterygius extremus* within an Apex Predator guild if a 50% completeness threshold is applied to the raw data, however (see Fig. S3). Within the Apex Predator guild, *Brachypterygius extremus, Pervushovisaurus bannovkensis* and *Pervushovisaurus campylodon* form a cluster. Confidence values are slightly increased in the new version of the cluster dendrogram, with an average bootstrap of 0.148 (vs 0.122 in (Fischer et al., 2016)) and an average approximate unbiased *P* value of 0.989 (vs 0.982 in Fischer et al. (2016)).

## Discussion

**Generic attribution of large Albian-Cenomanian platypterygiines**—The type material of *Platypterygius platydactylus* and *Ichthyosaurus campylodon* are barely overlapping, precluding an unambiguous referral to that genus. At the current state of knowledge, ‘*Platypterygius*’ *australis* and ‘*Platypterygius*’ *campylodon* do not share apomorphies; their rostral and dental similarities are plesiomorphic for platypterygiinae (Fischer et al., 2012). Most importantly, two peculiar features of *Ichthyosaurus campylodon* are shared with *Pervushovisaurus bannovkensis*: the prominent ridges forming 90° angles formed by the root cement in middle jaw/snout teeth and the ventrally protruding splenials. The type and only specimen *Pervushovisaurus bannovkensis* also exhibits a slight overbite (Fischer et al., 2014a), but the absence of teeth *in situ* precludes an unambiguous assessment of this feature in that taxon. Other differences between *Pervushovisaurus bannovkensis* and the syntypic material of *Ichthyosaurus campylodon* are the relatively smaller teeth in *Pervushovisaurus bannovkensis*, despite a seemingly larger skull size. The presence or absence of the other autapomorphic features of *Pervushovisaurus bannovkensis* cannot be assessed on material presently available of *I. campylodon*. Because of the similarities between *Pervushovisaurus bannovkensis* and *I. campylodon*, I propose to refer the species *I*. *campylodon* to the genus *Pervushovisaurus*. While additional specimens are certainly required to better assess whether *Pervushovisaurus campylodon* and *Pervushovisaurus bannovkensis* are conspecific or not, this is another important step in the clarification of Cretaceous ichthyosaur taxonomy.

Because *Platypterygius* as traditionally conceived is a wastebasket taxon, incorporating taxa distantly related to the Aptian type species *Platypterygius platydactylus*, assigning Cretaceous specimens to this genus, by default is not advisable (Fischer et al., 2016). However, the genus-group name *Myopterygius* Huene, 1922 is available. It was erected for a series of species: *Ichthyosaurus campylodon*, *Ichthyosaurus strombecki* (=nomen dubium Fischer et al., 2016), *Ichthyosaurus hildesiensis* (=nomen dubium Fischer et al., 2016), *Ichthyosaurus kokeni* (here regarded as Ophthalmosaurinae indet. see above), *Ichthyosaurus indicus* (=nomen dubium Fischer et al., 2016) and *Ichthyosaurus marathonensis* (=*Ichthyosaurus australis* (see Zammit, 2010)). There are thus two remaining candidates for the type species of *Myopterygius*: *I*. *campylodon* and *I. marathonensis* (= ‘*Platypterygius*’ *australis*). But there are no systematic rules regarding the designation of originally included nominal type species; the ICZN lists rules and best practices in Recommendations 69A.1–10.

On one hand, the species *Ichthyosaurus campylodon* is the first one on the list of species originally referred to *Myopterygius*. Before proposing the name *Myopterygius*, Huene (1922: 98) refers to the aforementioned species as the “Campylodongruppe” of Lydekker, reinforcing the idea that he probably intended *Ichthyosaurus campylodon* to be the equivalent of a type species for the genus *Myopterygius*. Resurrecting *Myopterygius* for reception of *Ichthyosaurs campylodon* would thus match the original interpretation of Huene, in a binomial that is still abundantly found in several museum collections across Europe. Such a move would match recommendations 69A.7, 69A.8, 69A.9, 69A.10 of the ICZN code, because *I. marathonensis* was poorly known when Huene published his work.

On the other hand, the species ‘*Platypterygius*’ *australis* is now known by abundant, excellently preserved material (Wade, 1984; Wade, 1990; Kear, 2005; Zammit, Norris & Kear, 2010) and could thus better positioned to fix an important genus-rank name (ICZN recommendation 69A.1). Currently, the number of specimens referred to as ‘*Platypterygius*’ *campylodon* is much larger than those referred to as ‘*Platypterygius*’ *australis*, but the novel features found in the syntypic series of ‘*Platypterygius*’ *campylodon* might result in a smaller number of specimens referable to this species.

Two additional factors need be considered here: the similarity between *Ichthyosaurus campylodon* and *Pervushovisaurus bannovkensis*, which indicate congeneric relationship between these two taxa and the unclear phylogenetic relationships among platypterygiine ichthyosaurs with subdivided nares (compare Fischer et al., 2014a; Fischer et al., 2016; Maxwell et al., 2015). This leaves two distinct solutions: (i) transfer *I. campylodon* and *Pervushovisaurus bannovkensis* to *Myopterygius*, with *I. campylodon* as the type species, and declare *Pervushovisaurus* as a junior synonym of *Myopterygius*. The status and generic attribution of ‘*Platypterygius*’ *australis* would be left undecided until a comprehensive study on the relationships of that taxon is undertaken, possibly needing a new genus-rank name for that taxon. (ii) Move *Ichthyosaurus campylodon* to *Pervushovisaurus* and leave the status of both *Myopterygius* and ‘*Platypterygius*’ *australis* undecided until further study on this later taxon. In order to move forward and stabilise the complex taxonomy of Cretaceous ichthyosaurs, I opt here of the second solution, which leaves the possibility to resurrect *Myopterygius* with ‘*Platypterygius*’ *australis* as its type species, but such a decision is beyond the scope of this paper.

**Figure 5:**
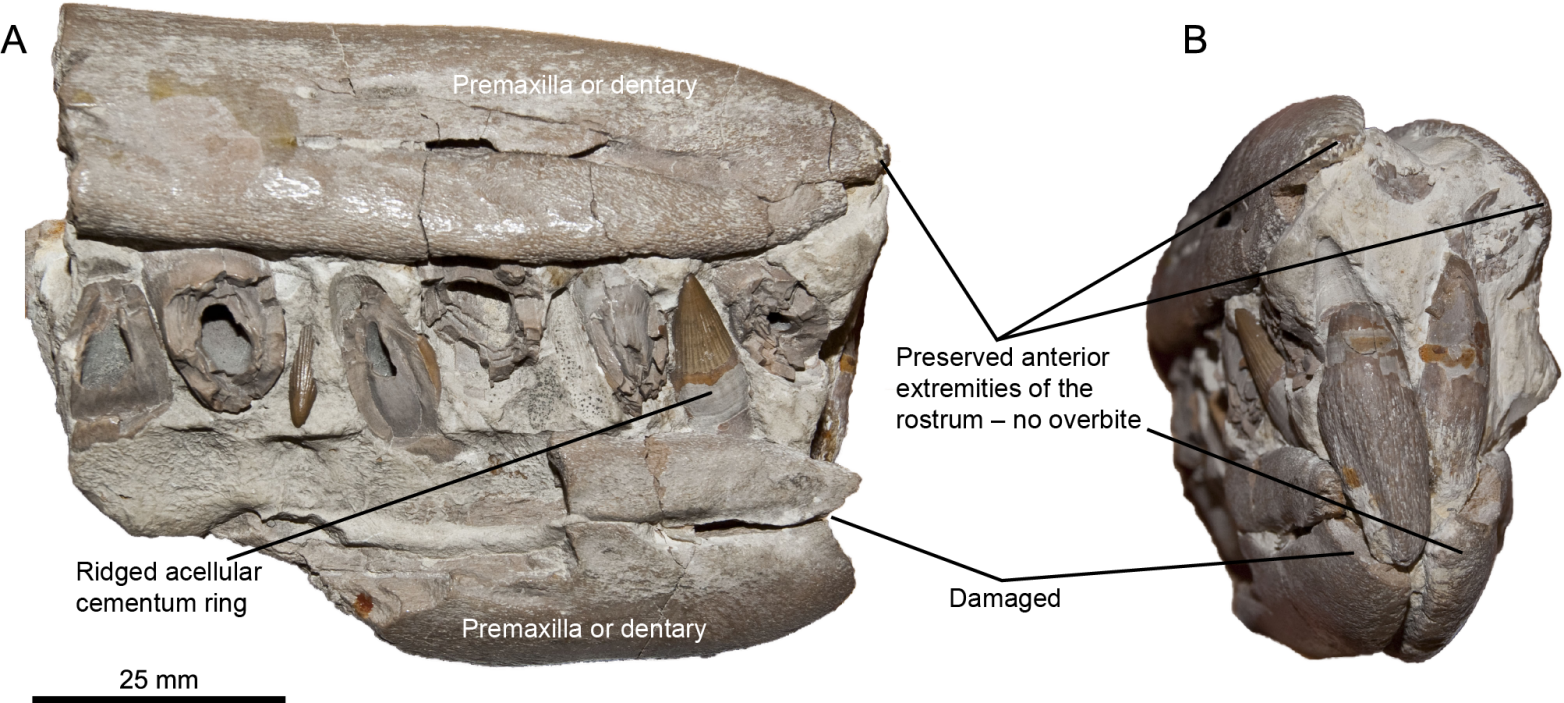
Possible second taxon in the Grey Chalk Subgroup. (A) Right lateral view. (B) Anterolateral view. Note the lack of a premaxillary overbite, as opposed to *Pervushovisaurus campylodon*, but the otherwise very similar teeth and rostrum shape, suggesting a similar ecological niche.

**The diversity of the last European ichthyosaurs**—The results from the cluster dendrogram analysis refine the claim for the presence of diversified ichthyosaur ecomorphs during the Early/earliest Cenomanian, as ‘*Platypterygius*’ *americanus* carries the Generalist guild up to the Early Cenomanian, even though the end its biozone is poorly constrained. *Pervushovisaurus campylodon* and *Pervushovisaurus bannovkensis*, two of the last ichthyosaurs, are tightly clustered within the Apex predator guild. Numerous other ichthyosaur specimens are present in the Grey Chalk Subgroup collections of the CAMSM and NHMUK (excluding the Cambridge Greensand member). These remains—mainly isolated teeth, centra and some basicranial bones—are compatible with derived platypterygiines and resemble ‘*Platypterygius*’ *hercynicus* (Kuhn, 1946; Kolb & Sander, 2009; Fischer, 2012), although with a slightly larger tooth size, and the specimen of ‘*Platypterygius*’ cf. *kiprijanoffi* described by Bardet (1989) from the Cenomanian of northwestern France. I have been unable to find other specimens that unambiguously possessed the unique dental and rostral features of *Pervushovisaurus* in the CAMSM, NHMUK and RBINS collections. There are two non-mutually exclusive reasons for this: (i) the prominent root ridges might be restricted to a small region of the snout and (ii) two weakly divergent platypterygiine species might be present in the Grey Chalk Subgroup. This latter possibility is exemplified by NHMUK 41367, a partial rostrum that lacks an overbite (Fig. 5), thus differing from the material hereby assigned to *Pervushovisaurus campylodon*. Because the overbite in *Pervushovisaurus campylodon* appears more strongly expressed in the smallest rostrum (CAMSM TN282) than in the largest (CAMSM B20671a), it is possible that this feature vary with ontogeny. It is however unlikely that this feature completely vanish with adulthood, because specimen CAMSM B20671a, which belong to end of the spectrum of parvipelvian skull size, still possesses a noticeable overbite. Sexual dimorphism is also a possibility, but it cannot be tested with the material currently at hand.

If present, any additional ichthyosaur species in the Grey Chalk Subgroup appear generally similar to *Pervushovisaurus campylodon* in terms of general tooth shape and inferred ecological niche. These taxa would fall within the ‘Apex predator’ niche, having absolutely large teeth and robust, relatively large, and heavily worn crowns (apex broken and polished). An example of intense wear can be seen on the rostrum CAMSM TN283 referred to Platypterygiinae indet. (Fig. 4B): one of the crowns has a significant portion of its apex spalled obliquely and polished. This is a rare wear stage for ichthyosaurs but common in so-called hypercarnivorous forms like the geosaurine metriorhynchid *Dakosaurus maximus* (Young et al., 2012) or tyrannosaurid theropods (Schubert & Ungar, 2005). Collectively, this suggests that *Pervushovisaurus* spp. and several coeval ichthyosaurs from the Cenomanian of western Europe occupied an apex predatory niche with a large body size, as indicated by isolated large centra and humeri in the CAMSM and NHMUK collections. The Cenomanian ichthyosaur record from the Grey Chalk Subgroup thus conforms to the global pattern of a two-step decline, ichthyosaurs being restricted to a single morphotype and ecological guild from the Early Cenomanian onwards: a large and long-snouted predator with robust teeth.

Yet, the small overbite in *Pervushovisaurus campylodon* raises questions regarding its function—if any. Moderate to large overbite evolved among leptonectid ichthyosaurs during the Early Jurassic (Huene, 1951; McGowan, 1986; McGowan, 1989; McGowan, 2003; Lomax, 2016). Overbite is not recorded in ichthyosaurs after the Toarcian; this feature thus re-evolved in *Pervushovisaurus campylodon* (or its ancestor if this feature is also present in *Pervushovisaurus bannovkensis*) after a 73 million years hiatus. A series of hypothetical functions of the sometimes extreme overbites seen in leptonectid ichthyosaurs have been made in the past (McGowan, 1979; Riess, 1986), including predatory (like a swordfish) and tactile (like a narwhal) functions (reviewed in Fischer, Guiomar & Godefroit, 2011). Leptonectids and *Pervushovisaurus campylodon* exhibit complex network of shallow grooves radiating from the anterior part of the fossa praemaxillaris, but such structure is also present in taxa with no overbite, such as *Suevoleviathan* (Maisch, 2001) and yet undescribed forms from France (V Fischer, pers. obs., 2012). These groove probably housed blood vessels, but their concentration in the rostral tip might suggest a sensory function, as in the recently described fossil phocoenid porpoise *Semirostrum cerutti*, which likely used its long dentary overbite to probe the sediment (Racicot et al., 2014). However, *Pervushovisaurus campylodon* clearly differ from the aforementioned taxa in having much stouter and larger rostrum and teeth and a much less conspicuous overbite, which might thus not yield any obvious functional advantage. Nevertheless, the presence of such a feature among Cretaceous ichthyosaurs illustrate the previously unappreciated phenotypic diversity of ichthyosaurs during this system.

##  Supplemental Information

10.7717/peerj.2604/supp-1 Supplemental Information 1 Supplementary information and figures

10.7717/peerj.2604/supp-2 Data S1 Ecomorphological dataset

## Institutional abbreviations

CAMSM: Sedgwick Museum of Earth Sciences, Cambridge University, Cambridge, UK
RBINS/IRSNB: Royal Belgian Institute of Natural Sciences, Brussels, Belgium
NHMUK: Natural History Museum, London, UK

## Additional Information and Declarations

### Data Availability

The following information was supplied regarding data availability: The raw data has been supplied as Supplementary File.

### New Species Registration

The following information was supplied regarding the registration of a newly described species: Publication LSID: urn:lsid:zoobank.org:pub:019DACEA-EBBE-4FAE-B885-3A9E5B1E1315.
