# Supplementary material for: Taxonomy of Platypterygius campylodon and the diversity of the last ichthyosaurs
Source: PeerJ. 2016 Oct 20;4:e2604. doi: 10.7717/peerj.2604 (PMC5075704; doi:10.7717/peerj.2604)
Supplement: Supplemental Information 1 [file peerj-04-2604-s001.docx]

**SUPPLEMENTARY INFORMATION**

**Taxonomy of *Platypterygius campylodon* and the diversity of the last ichthyosaurs**

Valentin Fischer

Evolution and Diversity Dynamics Lab, UR Geology, Université de Liège, Quartier Agora, 14 Allée du 6 Août, 4000 Liège, Belgium

[v.fischer@ulg.ac.be](mailto:v.fischer@ulg.ac.be)

**SUPPLEMENTARY PICTURES OF THE SYNTYPIC SERIES OF *ICHTHYOSAURUS* *CAMPYLODON* CARTER 1846**

**
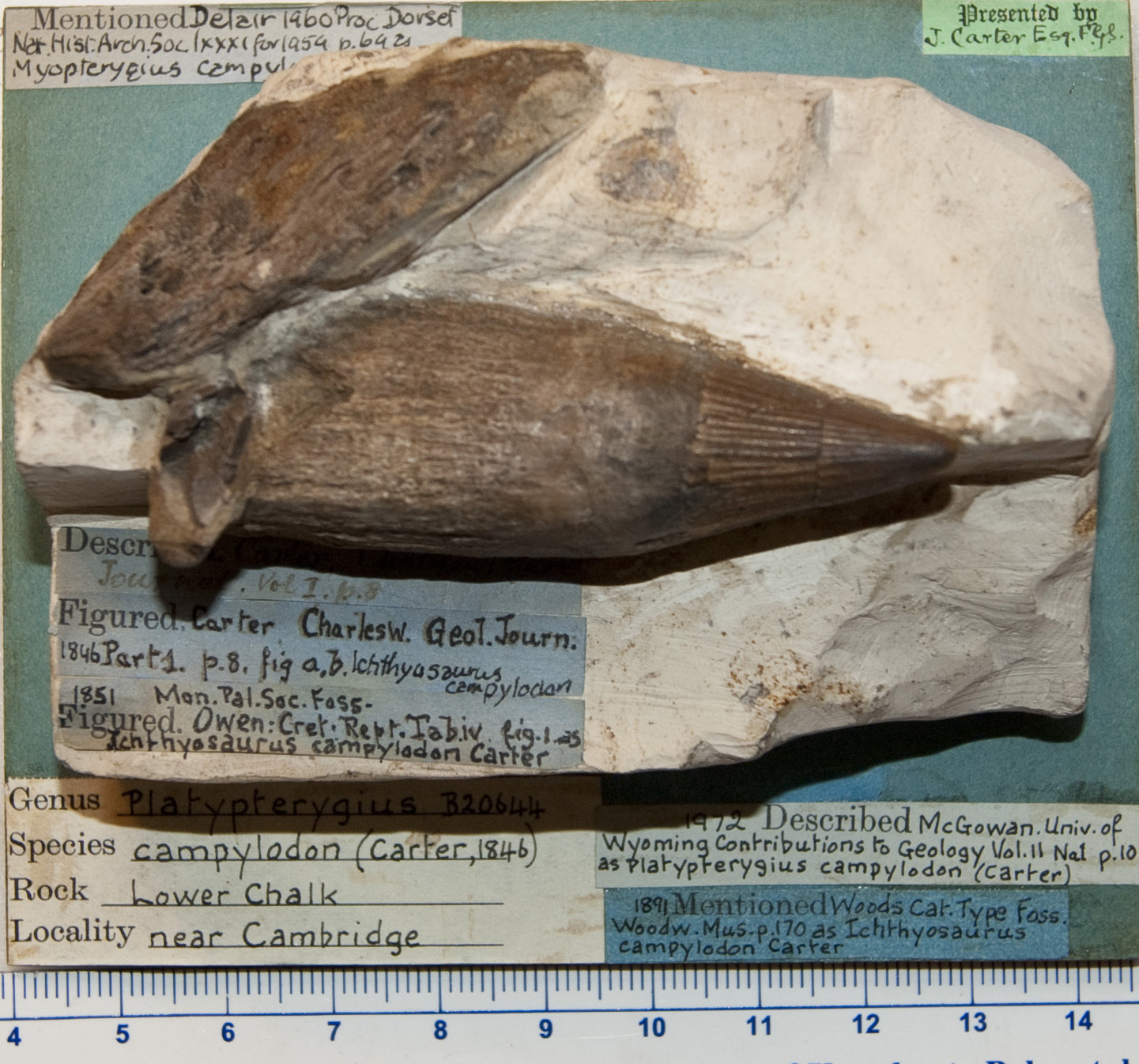
**

**Figure S.1. CAMSM B20644, with labels mentioning publications discussing or figuring this specimen, as well as the “Presented by J. Carter Esq. F.G.S.”, found in presumably all specimens from his collection and used in his description of *Ichthyosaurus* *campylodon*.**

**
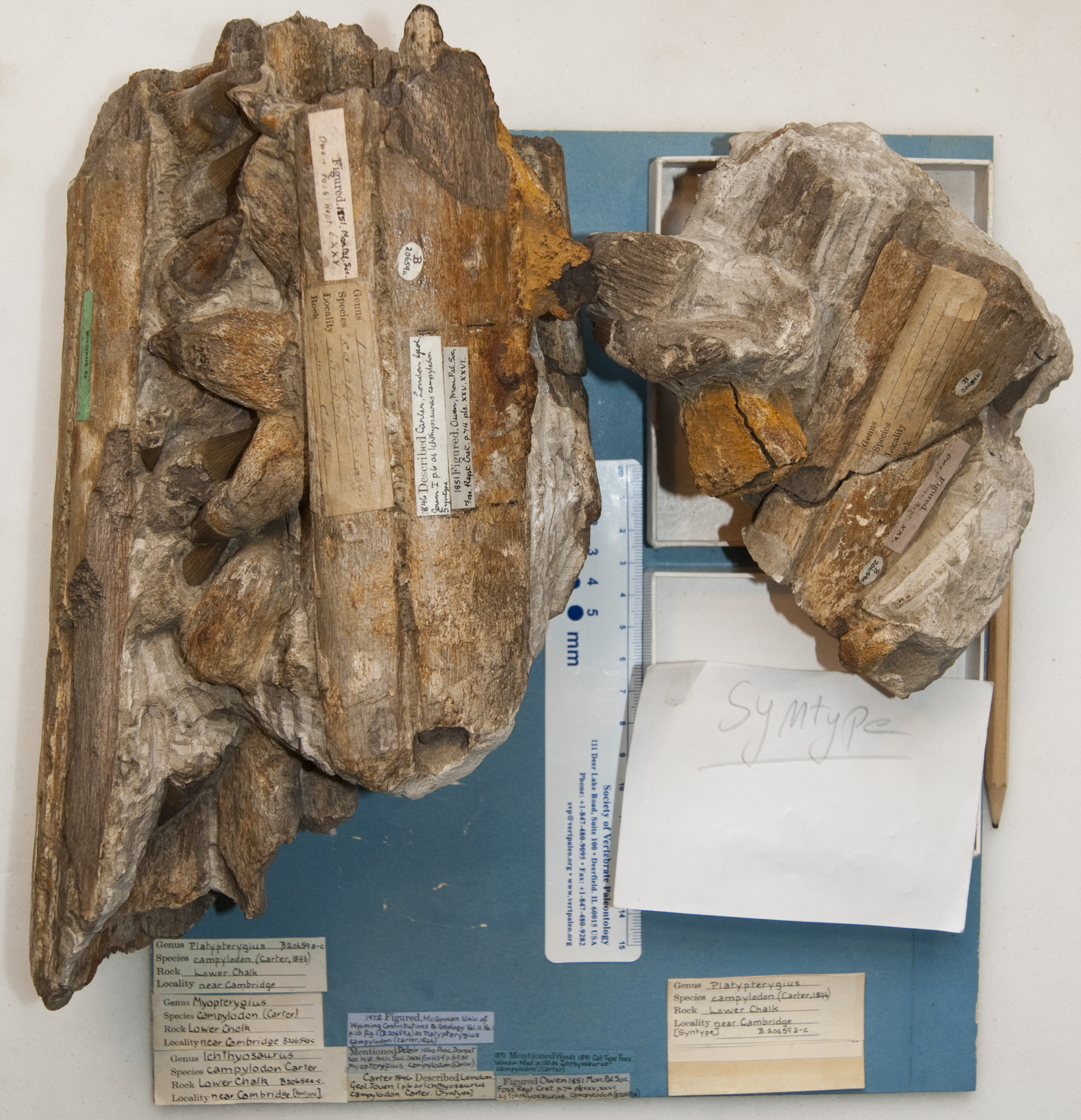
**

**Figure S.2. CAMSM B20659, the selected lectotype of *Pervushovisaurus campylodon*. with labels mentioning publications discussing or figuring this specimen, as well as the “Presented by J. Carter Esq. F.G.S.”, found in presumably all specimens from his collection and used in his description of *Ichthyosaurus* *campylodon*.**

**ECOLOGICAL DATA FOR CLUSTER DENDROGRAM ANALYSIS**

|  | Data sources | Tooth size | Crown shape | Crown relative size | Symphysis | Snout depth | Sclerotic aperture | Wear |
| --- | --- | --- | --- | --- | --- | --- | --- | --- |
| Ophthalmosaurus_icenicus | HM V1129 (Kirton, 1983) | 37.3 | 1.66 | 0.16 | 53.05 | 0.54 | 71.1 | NA |
| Ophthalmosaurus_natans | (Gilmore, 1905; McGowan, 1976); CM 603 | 29 | 2.35 | 0.14 | NA | 0.54 | 100 | NA |
| Mollesaurus_perialus | (Fernández & Talevi, 2014) | 20 | NA | 0.08 | NA | NA | 70.6 | NA |
| Acamptonectes_densus | GLAHM 132855 (*=SNHM1284-R) | NA | 2.66 | 0.17 | NA | 0.44* | NA | NA |
| Brachypterygius_extremus | (Kirton, 1983), CAMSMJ68516 | 53.4 | 1.54 | 0.26 | NA | 0.8 | NA | NA |
| Aegirosaurus_leptospondylus | (Bardet & Fernández, 2000); (*=RGHP LA 1) | 26* | 1.4* | NA | NA | 0.62 | 32.76 | 1.5* |
| Sveltonectes_insolitus | IRSNB R129 | 19 | 2.86 | 0.12 | 50.6 | 0.47 | 34.4 | 1.2 |
| Simbirskiasaurus_birjukovi | YKM 65119 | NA | 1.91 | 0.26 | NA | NA | NA | 2 |
| Platypterygius_australis | (Wade, 1990; Kear, Boles & Smith, 2003; Kear, 2005) | 55 | 1.65 | 0.31 | 40 | 0.48 | 31.5 | NA |
| Pervushovisaurus_bannovkensis | SSU 104a/24 | 60 | 1.49 | NA | NA | NA | NA | NA |
| Platypterygius_hercynicus | (Kuhn, 1946); MNHN2010 | 50 | 1.51 | 0.22 | NA | NA | NA | NA |
| Platypterygius_americanus | UW 2421 ((Romer, 1968) and photographs) | NA | 1.63 | 0.23 | 50.8 | 0.43 | 51 | NA |
| Platypterygius_sachicarum | DON-19671 ((Paramo, 1997) and photographs) | 40 | 1.53 | NA | NA | 0.49 | NA | 2.3 |
| Sisteronia_seeleyi | CAMSM TN1779; RGHP SI2 (*) | 33.8 | 1.75 | 0.2 | NA | NA | NA | 1.7* |
| Platypterygius_sp_Fr | RGHP PR1 | 55 | 1.91 | NA | NA | NA | NA | 2.4 |
| Pervushovisaurus_campylodon | CAMSM B20644; CAMSM B20671a (*) | 64.8 | 1.50 | NA | NA | NA | NA | 2.5* |

The values are rounded to the nearest % for visual purposes; the precise values can be found in attached text file “ecodata_mod2.txt”.

**Note on tooth wear quantification**

I used articulated rostra to count the relative occurrence of three stages of wear that I defined qualitatively as follows (as in Fischer et al., 2016): (i) no wear, the crown apex is pointed and still possesses its enamel microtexture; (ii) slight wear, the crown apex is rounded and the microtexture of the enamel is lost; (iii) intense wear, the crown apex is broken and/or spalled and this section is polished and smoothed by further food processing, so that we are confident this feature is not diagenetic or due to preparation damage. We gave a weight to each category (1, 2, 3 respectively) and quantified wear as the relative proportion of each wear stage multiplied by its weight.

**Ecological metrics employed**

1. Absolute tooth size
   1. Mid-rostrum tooth, total apico-basal size
   2. In mm; e.g. 55
2. Crown shape ratio
   1. Crown apicobasal height divided by crown basal diameter (at the base of enamel covering)
   2. E.g. 1.65
3. Crown relative size
   1. Crown apicobasal height divided by basioccipital diameter (which is a good proxy for intraquadrate length/gullet size)
   2. E.g. 0.304
4. Relative symphysial length
   1. Symphysis length divided by mandible length
   2. In %, e.g. 41
5. Relative snout depth (McGowan, 1976)
   1. Snout depth at midpoint divided by jaw length
   2. E.g. 0.484
6. Absolute sclerotic aperture
   1. Diameter of the aperture (=inner opening) of the sclerotic ring
   2. In mm; e.g. 31.5
7. Tooth wear
   1. Assign a weight to each wear stage of each functional (=fully erupted) crown (see comment above)
   2. Value is the sum of % of each stage; e.g. 0.5*1+025*2+0.25*3=1.75

**CLUSTER DENDROGRAM WITH 50% COMPLETENESS THRESHOLD**

**Figure S.3. Cluster dendrogram resulting from the analysis of the updated ecological data set, after applying a 50% completeness threshold on the raw data. The three main guilds remain present, but ‘*Platypterygius*’ *americanus* and ‘*Platypterygius*’ *hercynicus* form a clade within the Apex Predator guild, as in Fischer et al. (2016). Approximate unbiased *P-*values (red) and bootstrap values (green) are indicated at each node.**

**SUPPLEMENTARY REFERENCES**

Bardet N., Fernández M. 2000. A new ichthyosaur from the Upper Jurassic lithographic limestones of Bavaria. *Journal of Paleontology* 74:503–511.

Broili F. 1908. Ichthyosaurierreste aus der Kriede. *Neues Jahrbuch für Mineralogie, Geologie und Paläontologie. Beilage* 25:422–442.

Fernández M., Talevi M. 2014. Ophthalmosaurian (Ichthyosauria) records from the Aalenian–Bajocian of Patagonia (Argentina): an overview. *Geological Magazine* 151:49–59. DOI: 10.1017/S0016756813000058.

Fischer V., Maisch MW., Naish D., Kosma R., Liston J., Joger U., Krüger FJ., Pérez JP., Tainsh J. 2012. New ophthalmosaurid ichthyosaurs from the European Lower Cretaceous demonstrate extensive ichthyosaur survival across the Jurassic-Cretaceous boundary. *PLoS ONE* 7:e29234. DOI: 10.1371/journal.pone.0029234.

Fischer V., Bardet N., Benson RBJ., Arkhangelsky MS., Friedman M. 2016. Extinction of fish-shaped marine reptiles associated with reduced evolutionary rates and global environmental volatility. *Nature communications* 7:1–11. DOI: 10.1038/ncomms10825.

Gilmore CW. 1905. Osteology of *Baptanodon* (Marsh). *Memoirs of the Carnegie Museum* II:77–129.

Kear BP. 2005. Cranial morphology of *Platypterygius longmani* Wade, 1990 (Reptilia: Ichthyosauria) from the Lower Cretaceous of Australia. *Zoological Journal of the Linnean Society* 145:583–622.

Kear BP., Boles WE., Smith ET. 2003. Unusual gut contents in a Cretaceous ichthyosaur. *Proceedings of the Royal Society of London B Biological Sciences* 270:S206–S208.

Kirton AM. 1983. A review of British Upper Jurassic ichthyosaurs. Thesis Thesis. Newcastle upon Tyne: University of Newcastle upon Tyne.

Kuhn O. 1946. Ein skelett von *Ichthyosaurus hercynicus* n. sp. aus dem Aptien von Gitter. *Berichte der Naturforschenden Gesellschaft Bamberg* 29:69–82.

McGowan C. 1976. The description and phenetic relationships of a new ichthyosaur genus from the Upper Jurassic of England. *Canadian Journal of Earth Sciences* 13:668–683.

Paramo ME. 1997. *Platypterygius sachicarum* (Reptilia, Ichthyosauria) nueva especie del Cretácio de Colombia. *Revista Ingeominas* 6:1–12.

Romer AS. 1968. An ichthyosaur skull from the Cretaceous of Wyoming. *Contributions to Geology, Wyoming University* 7:27–41.

Wade M. 1990. A review of the Australian Cretaceous longipinnate ichthyosaur *Platypterygius* (Ichthyosauria, Ichthyopterygia). *Memoirs of the Queensland Museum* 28:115–137.
